# Proteomics risk scores and mortality in heart failure: Generalizability across populations

**DOI:** 10.1371/journal.pone.0350697

**Published:** 2026-06-23

**Authors:** Joseph J. Shearer, Jungnam Joo, Carolina G. Downie, Kayode O. Kuku, Maryam Hashemian, Eric Leifer, Suzette J. Bielinski, Véronique L. Roger

**Affiliations:** 1 Heart Disease Phenomics Laboratory, Epidemiology and Community Health Branch, National Heart, Lung, and Blood Institute, National Institutes of Health, Bethesda, Maryland, United States of America; 2 Office of Biostatistics Research, National Heart, Lung, and Blood Institute, National Institutes of Health, Bethesda, Maryland, United States of America; 3 Division of Epidemiology, Department of Quantitative Health Sciences, Mayo Clinic College of Medicine and Science, Rochester, Minnesota, United States of America; University of Naples Federico II, ITALY

## Abstract

**Background:**

Heart failure (HF) is a complex syndrome with high mortality. Proteomics risk scores have shown promise in predicting mortality beyond guideline-recommended clinical tools. It is crucial to understand how risk scores generated by different methods and populations perform, and whether they highlight the same protein targets relevant to outcomes.

**Methods:**

To examine whether the study design impacts proteomics scores designed to predict mortality in HF, we evaluated three published risk scores that used the SomaScan assay to measure plasma proteins in a community cohort, clinical trial, and registry. Each score was assessed in the aforementioned community cohort and Cox models examined the association of a 1-standard deviation increase in score with mortality, with and without adjustment for clinical covariates. Performance of each risk score to predict 5-year mortality risk was assessed using calibration plots and time-dependent area under the curve and compared with a clinical model.

**Results:**

Risk scores were similarly distributed and moderately correlated (Pearson correlation coefficient = 0.59–0.76). A 1-standard deviation increase in each risk score was associated with an increased risk of all-cause mortality: community cohort (HR = 2.70, 95% CI: 2.50–2.91); clinical trial (HR = 1.76, 95% CI: 1.65–1.88); registry (HR = 1.70, 95% CI: 1.6–1.81). Risk remained after adjustment for clinical covariates, although slightly attenuated, and similar across different ejection fraction categories. All risk scores showed strong calibration across the risk levels, alone, with an average expected over observed ratio ranging between 0.96–1.56. Seven proteins were included in at least two risk scores, with renin being included in all three.

**Conclusions:**

All three proteomics risk scores improved risk stratification in HF patients beyond guideline recommended clinical tools, independent of study design and ejection fraction. These results demonstrate that proteomics risk scores can enhance risk stratification across the HF syndrome, even when derived from different methods and populations.

## Introduction

Heart failure (HF) is a complex syndrome associated with high mortality [1]. Despite promising advancements in treatment options, [2] improvements in mortality risk have rapidly eroded [3]. This has left the community with an urgent need for novel approaches for mortality risk stratification in HF that incorporate objectively measured biological features, going beyond current clinical classification systems, such as ejection fraction, which are subject to high measurement variability in a clinical setting [4] and do not fully account for the complexity of the HF syndrome [5].

The 2022 AHA/ACC/HFSA guidelines suggest integration of multi-omic measurements may be a tool to better understand the biological heterogeneity associated with the HF syndrome and improve prognosis [6]. With the introduction of high-throughput proteomic technologies, proteomics risk scores have shown promise in predicting mortality risk beyond guideline-recommended clinical tools in HF [7]. However to further evaluate the potential clinical relevance of proteomics risk scores, it is essential to evaluate if study design impacts the performance of scores designed to predict mortality and whether they identify similar protein targets for clinical relevance.

Therefore, our primary objective was to assess whether the original study design used to develop a given risk score influences its ability to predict mortality within a community-based HF cohort. Secondarily, we aimed to evaluate how these risk scores perform across different ejection fraction categories and various clinical outcomes, while incorporating guideline-recommended risk stratification examined-tools, [6] such as Meta-analysis Global Group in Chronic HF (MAGGIC) scores or natriuretic peptides. Finally, we investigated whether comparing proteomics risk scores can offer mechanistic insights into potential biological targets underlying mortality risk in HF.

## Methods

### Source population and proteomics risk scores derivation

Three proteomics risk scores were selected to be evaluated further in a HF community cohort. Each of these scores was originally derived using unique study designs, including the aforementioned community cohort (Kuku 2024), [8] a clinical trial (Zhang 2022), [9] and a registry (Gui 2021) [10]. To be considered for the study the risk score needed to have met the following criteria: 1) HF population; 2) death was an outcome; 3) proteomic measures were collected using the SomaScan platform; and 4) coefficients were available or were able to be calculated for individual components of the proteomics risk score.

### Study population

All three risk scores were evaluated in the HF community cohort population (Kuku 2024), which has been previously described [8]. In brief, the HF community cohort was derived from the record linkage system of the Rochester Epidemiology Project, which captures nearly all clinical diagnoses, procedures, results, and outcomes in its catchment area [11,12]. Natural language processing was used to identify HF patients 20 years or older from southeastern Minnesota [13] and research nurses validated the HF diagnosis using the Framingham criteria [14]. Patients were approached in the hospital or after an outpatient encounter to provide written consent between 2003 and 2012. The Mayo Clinic and Olmsted Medical Center Institutional Review Boards approved this study.

### Proteomic measurements and risk scores calculations

Details of the SomaScan assay technology have been described previously [15–17]. Briefly, SomaScan uses slow off-rate chemically modified single-stranded DNA aptamer reagents (SOMAmer), which are short oligonucleotides capable of binding target proteins or peptides with high specificity and affinity. Each risk score was calculated in the community cohort population as a summation of the measured SOMAmer expression multiplied by the beta-coefficient (S1 Fig in S1 File). The community cohort and clinical trial proteomics risk scores used a standardized log-transformed expression, and the registry risk score used an unstandardized log-transformed expression. The registry risk score only provided the hazard ratio (HR) of the multiprotein Cox model, and therefore, we used the log-transformed HR as a beta-coefficient to calculate the proteomics risk score. Proteomics risk scores were then standardized by setting the mean to 0 and the standard deviation to 1.

### Outcome assessment

The primary outcome was all-cause mortality; however, HF hospitalization or cardiovascular death were considered as secondary outcomes since they were the primary endpoint used for the clinical trial risk score. Death information was obtained from the healthcare providers that participated in the Rochester Epidemiology Project, from Minnesota death certificates, and patient record linkage to the National Death Index Plus. All-cause and cardiovascular-related mortality (International Classification of Diseases, Tenth Revision, codes I00–178) were considered. Patients alive at the end of follow-up were censored as of March 31, 2021, or the date of last known healthcare contact, whichever was earlier.

### Statistical analysis

Pearson correlation coefficients were calculated between three risk scores and key clinical variables (age, N-Terminal pro-B Type Natriuretic Peptide [NT-proBNP] and MAGGIC score).To assess model performance, we fit a Cox proportional hazard model using a proteomics risk score as a predictor and examined the HR of each risk score in the community cohort population. We further investigated the performance of the risk scores for predicting 5-year outcome using discrimination-based time-dependent area under the curve (tAUC) and calibration by examining the observed and predicted event rates. For HF hospitalization or cardiovascular death, a cause-specific Cox model was used. The cumulative incidence was calculated by considering non-cardiovascular death as a competing risk. The model was further adjusted for the MAGGIC score and NTproBNP. Our adjustment strategy intentionally focused on MAGGIC and NT-proBNP, both of which are, prognostic indicators of risk in HF, which are well validated and recommended by the HF guidelines. This approach was selected to minimize overfitting and multicollinearity [6].

### Common protein targets

We examined SOMAmers commonly included in three proteomics risk scores. We classified HF patients using kmeans clustering. Further, we calculated modified versions of each PRS by including only the SOMAmers that were included at least one of the other two risk scores.

## Results

**Fig 1:**
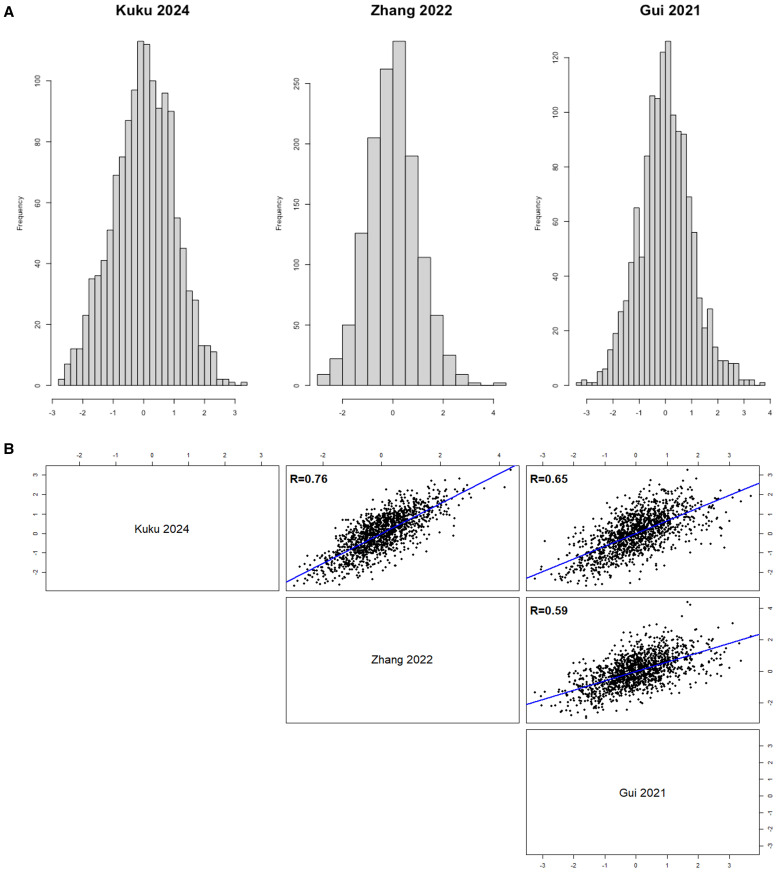
Proteomics risk score distribution (A) and correlations (B) within community cohort.

Characteristics of the three risk score source populations are summarized in Table 1, including a community cohort, a clinical trial, and a registry. All three studies measured proteomics in participants with HF using the SomaScan assay by SomaLogic. The risk scores were developed and validated in populations that were largely advanced in age, male and White. Two studies only included patients with HF with reduced ejection fraction, while one included all HF cases regardless of ejection fraction. After standardization, the proteomics risk scores were similarly distributed and moderately correlated with one another (Pearson correlation coefficient = 0.59 to 0.76) when applied to the community HF cohort (Fig 1). Proteomics risk scores were generally more consistently correlated with one another than with established risk factors, such as age, NTproBNP, and the MAGGIC Score (S2 Fig in S1 File).

**Table 1:** Proteomics risk score study characteristics.

Author	Kuku 2024	Zhang 2022	Gui 2021
Source Population	Community Cohort	Clinical Trial	Registry
N^1^	885/496	1258/1257	681/336
Age^1^	Median 78 (IQR: 69, 85)/ 76 (IQR: 65–84)	Median 67 (IQR: 59,73) / 64 (IQR: 61,74)	Mean 68 (SD: 12)/ 68 (SD: 12)
Female (%)^1^	50/45	19/19	36/34
White (%)^1,2^	97/96	96/96	50/49
Ejection Fraction (%)^1,3^	All, median 55 (IQR: 35, 65) / 50 (IQR:33,61)	≤35, median 30 (IQR: 25,34) / 32 (IQR: 28, 35)	<50, mean 35 (SD: 11) / 35 (SD:11)
Biosample Type	Plasma	Serum	Plasma
SomaScan Platform	7K	5K	5K
Protein Targets Measured (N)	7289	4076	4453
Transformation and Standardization Approach for SOMAmer Measurements	Log2 transformed and standardized	Log2 transformed and standardized	Natural log transformed without standardization
Statistical Approach for Deriving Risk Score	LASSO penalized regression	LASSO penalized regression	Lasso-penalized Cox regression
Primary Outcome	All-cause mortality	Heart failure hospitalization or cardiovascular mortality	All-cause mortality
Number of Protein Targets Included in Score	39	64 (57 available for analysis)^4^	8

^1^Descriptive statistics separated by discover and validation cohorts.

^2^Defined as European American (Gui, 2021).

^3^Values state the study inclusion criteria, followed by the study estimate.

^4^Only 57 of the 64 PRS scores were available for analysis in the community cohort due to quality control.

### Incremental value and predictive performance of proteomics risk scores

Among the 1,351 patients with proteomics enrolled in the HF community cohort, the 10-year outcome rate of the primary outcome of all-cause mortality was 75%, and the secondary outcomes of cardiovascular-related mortality was 38%, HF hospitalization was 37%, and HF hospitalization or cardiovascular-related mortality was 56%. A 1-standard deviation increase in each risk score was associated with a large increased risk of all-cause mortality: community cohort (HR = 2.70, 95% CI: 2.50–2.91); clinical trial (HR = 1.76, 95% CI: 1.65–1.88); registry (HR = 1.70, 95% CI: 1.60–1.81) (Table 2). The increased risk was slightly attenuated for all-cause mortality with adjustment for clinical covariates and was similar across ejection fraction categories and for the secondary outcomes of cardiovascular-related mortality and HF hospitalization or cardiovascular-related death (S1 and S2 Tables in S1 File).

**Table 2:** All-cause mortality risk in the community cohort, by proteomics risk score.

Model	Kuku 2024	Zhang 2022	Gui 2021
**Overall (N = 1351)** ^**1**^			
Crude	2.70 (2.50, 2.91)	1.76 (1.65, 1.88)	1.70 (1.60, 1.81)
MAGGIC-adjusted	2.38 (2.18, 2.59)	1.49 (1.39, 1.60)	1.54 (1.44, 1.64)
MAGGIC + NT-proBNP-adjusted	2.40 (2.18, 2.64)	1.40 (1.28, 1.53)	1.46 (1.36, 1.57)
**HFrEF (N = 415)**			
Crude	2.77 (2.40, 3.20)	1.73 (1.54, 1.94)	1.64 (1.47, 1.84)
MAGGIC-adjusted	2.42 (2.02, 2.90)	1.41 (1.24, 1.62)	1.40 (1.23, 1.59)
MAGGIC + NT-proBNP-adjusted	2.41 (1.98, 2.94)	1.30 (1.11, 1.53)	1.33 (1.16, 1.52)
**HFpEF (N = 912)**			
Crude	2.65 (2.42, 2.90)	1.80 (1.66, 1.95)	1.72 (1.60, 1.86)
MAGGIC-adjusted	2.33 (2.11, 2.58)	1.55 (1.42, 1.68)	1.58 (1.46, 1.71)
MAGGIC + NT-proBNP-adjusted	2.36 (2.09, 2.66)	1.40 (1.25, 1.56)	1.48 (1.36, 1.61)

Values are hazard ratios and 95% confidence intervals per 1 standard deviation increase in score.

^1^Missing ejection fraction information (N = 24).

**Fig 2:**
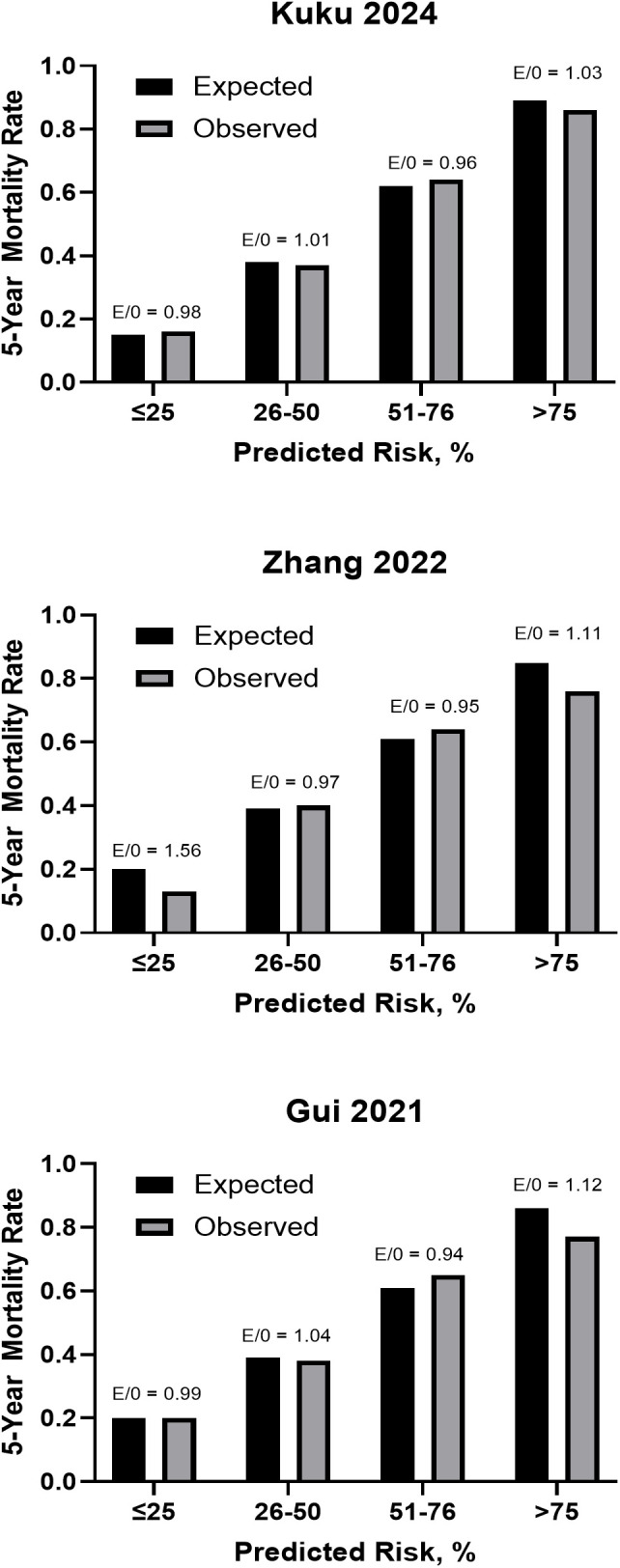
Calibration plot for 5-year mortality risk in community cohort by risk score.

**Fig 3:**
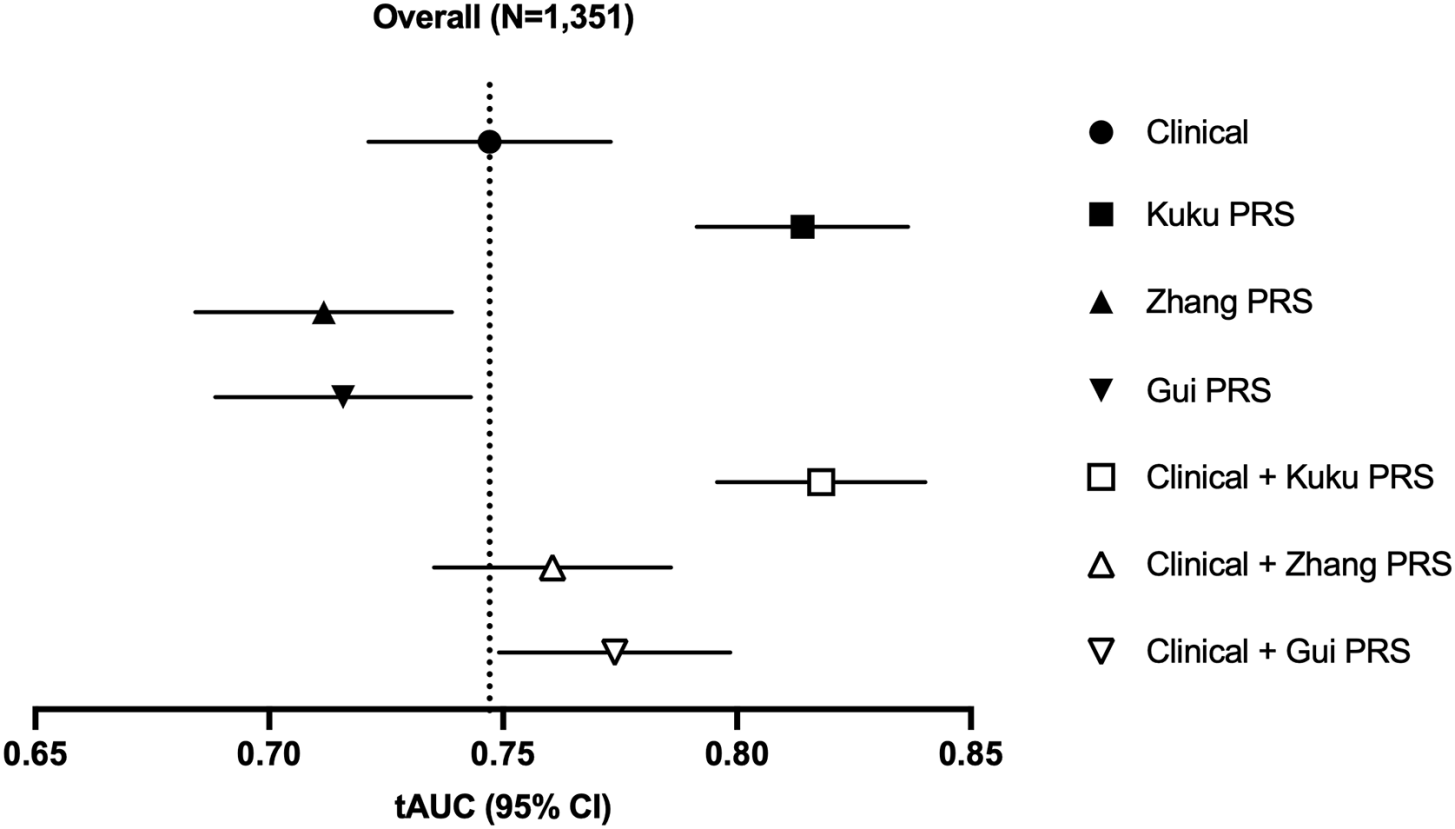
Time-dependent AUC of 5-year mortality risk in community cohort by risk score.

All three risk scores showed strong calibration across the entire risk spectrum, alone, with an average expected over observed ratio ranging between 0.96 and 1.56 (Fig 2 and S3 Fig in S1 File). All three risk scores also improved predictive performance of the model beyond clinical variables alone (S4 Fig in S1 File), across the entire risk spectrum, evident by the improvement in tAUC (Fig 3). Findings were consistent across ejection fraction categories (S5 Fig in S1 File)

### Protein targets across published proteomics risk scores

**Fig 4:**
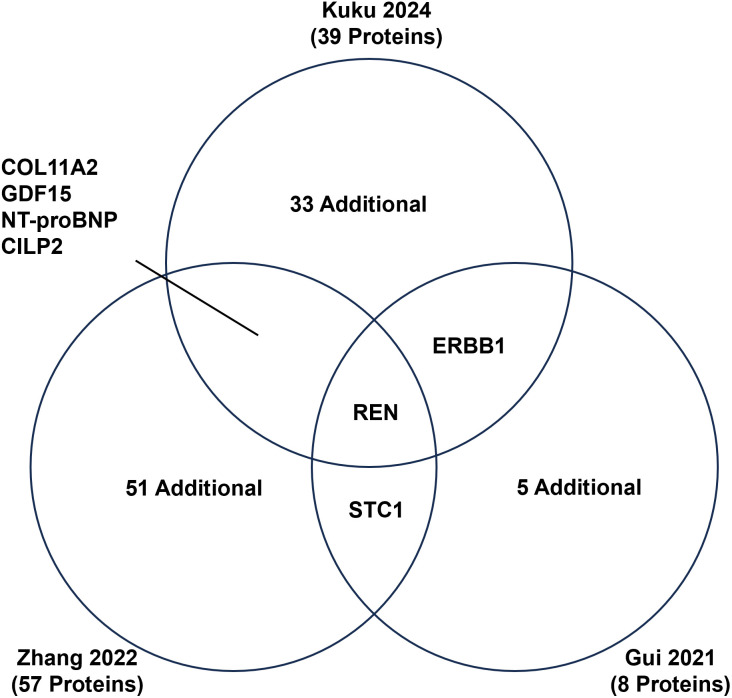
Common protein targets across risk scores.

The number of proteins included in each risk score ranged between 8 and 57. Seven proteins were included in at least two scores, with renin being the only protein included in all three (Fig 4). Unsupervised clustering analyses based on expression of the seven common protein targets, showed strong mortality risk stratification (S6 Fig in S1 File). When proteomics risk score was calculated using only SOMAmers included in at least two risk scores, the number of SOMAmers was reduced (6 for community cohort and clinical trial, and 3 for registry). Despite this, the HR per 1-standard deviation increase remained high (S3 Table in S1 File).

As the partial overlap between the derivation and validation samples (855 of 1,351 patients) could introduce optimism bias, we repeated the analysis within the validation dataset of the community cohort. The performance of the community cohort PRS showed minor attenuation, and all models demonstrated results remained consistent with our primary findings, while exhibiting some reduction in statistical significance due to the smaller sample size as expected.

## Discussion

We found that all three proteomics risk scores significantly enhanced risk stratification in a community-based HF cohort, outperforming standard guideline-recommended clinical tools [6]. Notably, these improvements were consistent despite the scores being developed in diverse HF populations, including a registry, clinical trial, and community cohort. Importantly, the scores demonstrated robust performance regardless of ejection fraction and identified renin as a shared protein target. These findings underscore the strong potential of proteomics to not only refine risk prediction in HF but also to advance our understanding of the biological mechanisms underlying this risk.

### Previous studies of proteomics of HF

High-throughput proteomics have emerged as a potential tool for improving heart failure risk stratification, including the development of risk scores; [7] however, the large heterogeneity in study design, proteomic technology, and reporting has hindered our ability to generate generalizable inferences suitable for clinical application.

We previously demonstrated the incremental value of a proteomics risk score in HF risk prediction within a community cohort, beyond currently guideline recommended tools and regardless of ejection fraction [8]. While that study followed Transparent Reporting of a multivariable prediction model for Individual Prognosis or Diagnosis (TRIPOD) guidelines [18] to optimize reproducibility and validity of the risk score, the lack of external community HF cohorts with rich clinical phenotyping and proteomic measures, and HF-specific proteomics risk scores limited our ability to fully assess their robustness as a clinical tool for HF risk prediction. In an attempt to overcome this limitation, we examined two additional published studies most similar to ours with available information on proteomic measurements. This created an opportunity to assess how proteomics risk scores derived using diverse study designs would perform in a community cohort.

Zhang et al. [9] used data from two clinical trials comprised of individuals with reduced ejection fraction for their discovery and replication of their risk score. The primary outcome of their risk score was a composite outcome to evaluate cardiovascular death or HF hospitalization but also evaluated all-cause mortality as a secondary outcome. Results were similar when restricted to SOMAmers selected for all-cause mortality in their study. While a clinical trial is considered the gold standard design to establish a causal treatment effect, the population enrolled often does not represent the entire HF syndrome population observed in clinical practice, due to the trial’s inclusion and exclusion criteria [19].

The study by Gui et al. [10] included individuals with reduced ejection fraction from a HF registry. Despite similar concerns of selection bias associated with registries, the study was able to demonstrate the incremental predictive value of their risk score and all-cause mortality, beyond MAGGIC and NT-proBNP. A major strength of Gui et al. was that, unlike the other two studies, their risk score was developed in a racially and ethnically diverse cohort, who are known to experience higher levels of HF mortality [20].

By examining proteomics risk scores developed using different study designs with different potential biases in a common community cohort, we provided further evidence for the clinical relevance of proteomics in HF risk prediction. Importantly, the proteomic scores outperformed recommended risk stratification tools and across ejection fraction groups.

### Specific protein targets and biological plausibility

Identifying common proteins across scores that were derived using different methods and study populations may provide some evidence for biological plausibility or clinical relevance warranting further investigation. We found seven proteins were included in at least two risk scores and that stratifying patients based on their expression of these seven proteins alone resulted in strong risk stratification. This may represent an informal approach at validation of protein targets.

Among the common protein targets, renin was the only protein included in all three scores. Renin is an enzyme produced by the kidney that activates the renin-angiotensin-aldosterone system, a key pathway in the pathogenesis of HF [21]. While not currently a guideline recommended biomarker in the management of HF, this result further demonstrates the importance of incorporating more robust metrics of kidney health in the prognosis in HF. This point was highlighted in the recent presidential advisory statement from the American Heart Association on the growing concerns of cardiovascular-kidney-metabolic syndrome [22] and increasing cardiorenal-related mortality in the United States [23].

In addition to renin, six other protein targets were identified in multiple proteomics risk scores (COL11A2, GDF15, NT-proBNP, CILP2, ERBB1, STC1). These included natriuretic peptide B, a well-established and guideline recommended prognostic biomarker in HF [24]. Identification of common protein targets derived from distinct source populations and statistical approaches provide more evidence for the biological plausibility of these findings and the need for further mechanistic evaluation.

Despite the observed added value of each risk score and a subset of common proteins, there was minimal overlap of all the proteins examined across the three scores. This highlights an important issue related to dimensionality reduction procedures, which may differ across studies leading to the retention of different proteins that still would fall under similar mechanistic pathways. Rather than emphasizing differences in the specific proteins retained by each score. The consistent risk prediction performance across studies strengthens the growing body of evidence supporting the value of proteomics for risk prediction in HF. These considerations underscore the need to harmonize methods for target identification in large-scale proteomic datasets, as well as the statistical strategies employed during dimensionality reduction—both of which can significantly influence clinical interpretations and downstream inferences.

### Clinical implications

Although high-throughput proteomics has emerged as a potential tool for improving heart failure risk stratification, its widespread clinical utility remains to be assessed. Proteomics provides the ability to evaluate thousands of interconnected proteins simultaneously to identify highly relevant protein targets. However, cost and clinical prediction robustness remain barriers to its widespread adoption and clinical use. Our results support that proteomic-based risk scores, derived from diverse source populations and study designs, have the potential to overcome the underperformance of many published risk scores in real-world settings [25,26]. Our results suggest that novel proteomic-based risk scores can offer robust, clinically meaningful improvements in risk stratification across the full spectrum of HF syndrome.

### Limitations and strengths

Our study has some limitations to consider. Two SomaScan versions were used across the three studies although the 7K platform contains the entire 5K platform [27]. Two of the study populations were predominantly White, potentially limiting the generalizability. We observed higher performance of the community cohort PRS when it was applied to the overall community cohort population. However, this was not surprising since the community cohort PRS was developed on 855 of the 1351 patients in the community cohort. Due to the limited sample size available for subgroup analyses, we decided to use all 1351 patients in the community cohort to evaluate each risk score. However, further validation of the community cohort’s risk score is needed in a completely independent population. The etiology of the HF syndrome is most often multifactorial. Because the etiology was not part of the original published scores, it was not incorporated into our analyses.

Our study has several notable strengths. Most importantly, proteomics risk scores were evaluated within a community-based cohort, reducing referral bias relative to clinical trial and registry populations. This advantage was maximized through linkage with the Rochester Epidemiology Project, which captures nearly all inpatient and outpatient encounters within a defined geographic region, providing exceptionally comprehensive clinical data and complete follow-up. In addition, we adjusted for key established prognostic variables, including NT-proBNP, MAGGIC, and ejection fraction, demonstrating the robustness and incremental clinical value of the proteomics risk scores beyond guidelines recommended tools [6]. All three cohorts were recruited prior to the introduction of angiotensin receptor–neprilysin inhibitors and sodium–glucose cotransporter 2 inhibitors, ensuring comparable therapeutic eras and minimizing confounding from evolving treatment practices. Together, these strengths support our conclusion that three risk scores consistently outperformed guideline-based risk stratification across study designs and ejection fraction categories, underscoring their generalizability and clinical relevance.

## Conclusion

All three proteomics risk scores significantly enhanced risk stratification in a heart failure (HF) community cohort, surpassing guideline-recommended clinical tools and performing independently of ejection fraction. Notably, renin emerged as the only protein consistently identified across all scores. These findings highlight the potential of proteomics risk scores to improve risk stratification across the entire HF spectrum, while offering valuable mechanistic insights, regardless of the diverse source populations and study designs from which they were derived.

## Supporting information

S1 File Proteomics risk score analyses in the community cohort. (DOCX)
