# Supplementary material for: Proteomics risk scores and mortality in heart failure: Generalizability across populations
Source: PLoS One. 2026 Jun 23;21(6):e0350697. doi: 10.1371/journal.pone.0350697 (PMC13289903; doi:10.1371/journal.pone.0350697)

# Supporting Information

**S1 Table.** Cardiovascular mortality risk in community cohort, by proteomics risk score

**S2 Table.** Heart failure hospitalization or cardiovascular mortality risk in community cohort, by proteomics risk score.

**S3 Table.** All-cause mortality risk in community cohort, by original proteomics risk score common SOMAmers in at least two scores.

**S1 Fig.** Linear predictor equations for proteomics risk scores.

**S2 Fig.** Correlations of proteomics risk scores with key heart failure risk factor.

**S3 Fig.** Calibration plot of 5-year mortality risk in community cohort by proteomics risk score stratified by ejection fraction.

**S4 Fig.** Time-dependent ROC curves of 5-year mortality in community cohort by proteomics risk score.(A) clinical, Kuku PRS and clinical + Kuku PRS, (B) clinical, Zhang PRS, clinical + Zhang PRS, (C) clinical, Gui PRS, clinical + Gui PRS.

**S5 Fig.** Time-dependent AUC of 5-year mortality risk in community cohort by proteomics risk score stratified by ejection fraction.

**S6 Fig.** Kaplan-Meier survival curves of three HF patient groups generated by K-means clustering using 7 common SOMAmers that were included in at least two of the three proteomics risk scores.

| **S1 Table. Cardiovascular mortality risk in community cohort, by protein risk score** | | | |
| --- | --- | --- | --- |
| **Model** | **Kuku 2024** | **Zhang 2022** | **Gui 2021** |
| **Overall (N=1351)** |  |  |  |
| Crude | 2.76 (2.48, 3.07) | 1.89 (1.73, 2.07) | 1.60 (1.47, 1.74) |
| MAGGIC-adjusted | 2.21 (1.96, 2.50) | 1.54 (1.40, 1.70) | 1.38 (1.26, 1.53) |
| MAGGIC + NT-proBNP-adjusted | 2.02 (1.76, 2.32) | 1.32 (1.17, 1.50) | 1.27 (1.15, 1.41) |
| **HFrEF (N=415)** |  |  |  |
| Crude | 2.87 (2.39, 3.45) | 1.83 (1.58, 2.12) | 1.65 (1.43, 1.91) |
| MAGGIC-adjusted | 2.34 (1.86, 2.94) | 1.47 (1.24, 1.75) | 1.37 (1.16, 1.62) |
| MAGGIC + NT-proBNP-adjusted | 2.24 (1.74, 2.87) | 1.33 (1.08, 1.63) | 1.28 (1.08, 1.52) |
| **HFpEF (N=912)** |  |  |  |
| Crude | 2.73 (2.40, 3.12) | 1.91 (1.71, 2.15) | 1.59 (1.42, 1.77) |
| MAGGIC-adjusted | 2.22 (1.91, 2.58) | 1.58 (1.40, 1.79) | 1.41 (1.25, 1.59) |
| MAGGIC + NT-proBNP-adjusted | 1.99 (1.66, 2.38) | 1.29 (1.09, 1.52) | 1.26 (1.11, 1.44) |
| Values are hazard ratios and 95% confidence intervals per 1 standard deviation increase in score | | | |
| Missing ejection fraction information (N=24) | | | |

| **S2 Table. Heart failure hospitalization or cardiovascular mortality risk in community cohort, by protein risk score** | | | |
| --- | --- | --- | --- |
| **Model** | **Kuku 2024** | **Zhang 2022** | **Gui 2021** |
| **Overall (N=1351)** |  |  |  |
| Crude | 2.00 (1.84, 2.18) | 1.76 (1.63, 1.89) | 1.45 (1.35, 1.55) |
| MAGGIC-adjusted | 1.67 (1.51, 1.84) | 1.54 (1.42, 1.67) | 1.29 (1.19, 1.39) |
| MAGGIC + NT-proBNP-adjusted | 1.41 (1.26, 1.57) | 1.29 (1.16, 1.44) | 1.18 (1.08, 1.28) |
| **HFrEF (N=415)** |  |  |  |
| Crude | 1.78 (1.54, 2.04) | 1.54 (1.37, 1.74) | 1.42 (1.26, 1.59) |
| MAGGIC-adjusted | 1.41 (1.18, 1.69) | 1.33 (1.16, 1.52) | 1.24 (1.09, 1.41) |
| MAGGIC + NT-proBNP-adjusted | 1.31 (1.07, 1.60) | 1.25 (1.06, 1.48) | 1.19 (1.04, 1.36) |
| **HFpEF (N=912)** |  |  |  |
| Crude | 2.21 (1.99, 2.46) | 1.86 (1.69, 2.04) | 1.50 (1.37, 1.64) |
| MAGGIC-adjusted | 1.91 (1.69, 2.16) | 1.64 (1.47, 1.82) | 1.34 (1.22, 1.48) |
| MAGGIC + NT-proBNP-adjusted | 1.60 (1.38, 1.85) | 1.33 (1.16, 1.53) | 1.19 (1.07, 1.33) |
| Values are hazard ratios and 95% confidence intervals per 1 standard deviation increase in score | | | |

| **S3 Table. All-cause mortality risk in community cohort, by original protein risk score common SOMAmers in at least two scores** | | | |
| --- | --- | --- | --- |
| **PRS** | **Model** | **Original** | **Common** |
| **Kuku 2024** | SOMAmers (N) | 39 | 6 |
|  | Crude | 2.70 (2.50, 2.91) | 1.90 (1.78, 2.03) |
|  | MAGGIC + NT-proBNP-adjusted | 2.40 (2.18, 2.64) | 1.54 (1.43, 1.67) |
| **Zhang 2022** | SOMAmers (N) | 57 | 6 |
|  | Crude | 1.76 (1.65, 1.88) | 1.88 (1.75, 2.01) |
|  | MAGGIC + NT-proBNP-adjusted | 1.40 (1.28, 1.53) | 1.63 (1.46, 1.83) |
| **Gui 2021** | SOMAmers (N) | 8 | 3 |
|  | Crude | 1.70 (1.60, 1.81) | 1.69 (1.59, 1.79) |
|  | MAGGIC + NT-proBNP-adjusted | 1.46 (1.36, 1.57) | 1.45 (1.36, 1.55) |
| Values are hazard ratios and 95% confidence intervals per 1 standard deviation increase in score | | | |

**S1 Fig.** Linear predictor equations for proteomics risk scores.


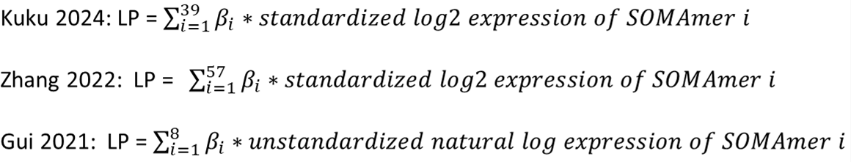


**S2 Fig.** Correlations of proteomics risk scores with key heart failure risk factor.


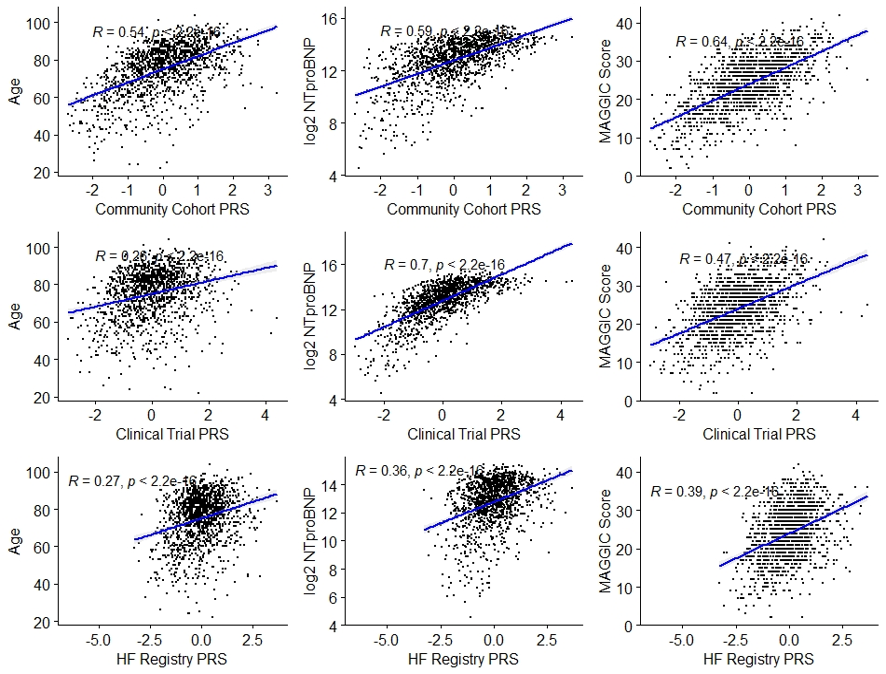


**S3 Fig.** Calibration plot of 5-year mortality risk in community cohort by proteomics risk score stratified by ejection fraction.

**
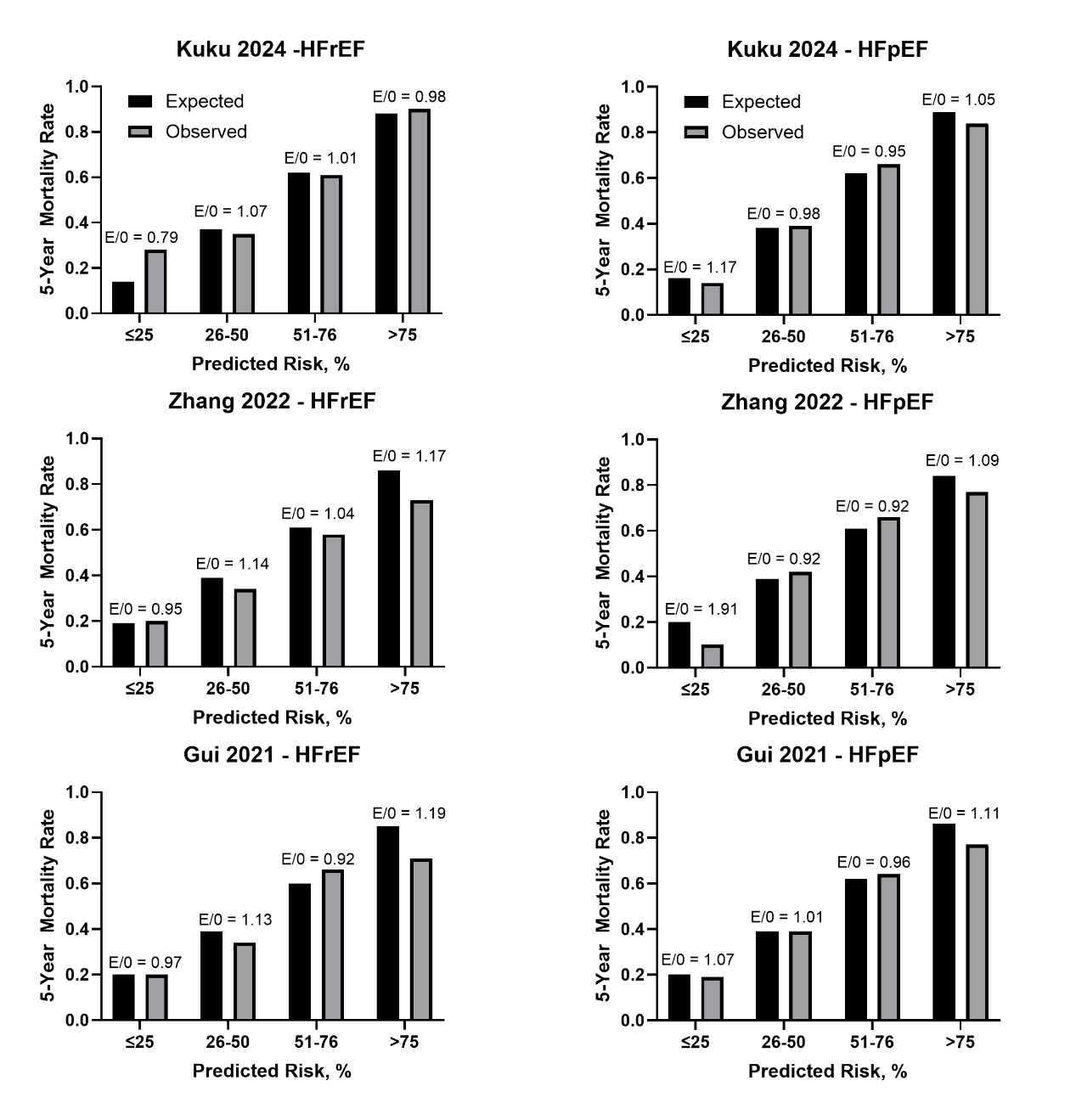
**

**S4 Fig.** Time-dependent ROC curves of 5-year mortality in community cohort by proteomics risk score.(A) clinical, Kuku PRS and clinical + Kuku PRS, (B) clinical, Zhang PRS, clinical + Zhang PRS, (C) clinical, Gui PRS, clinical + Gui PRS.

**
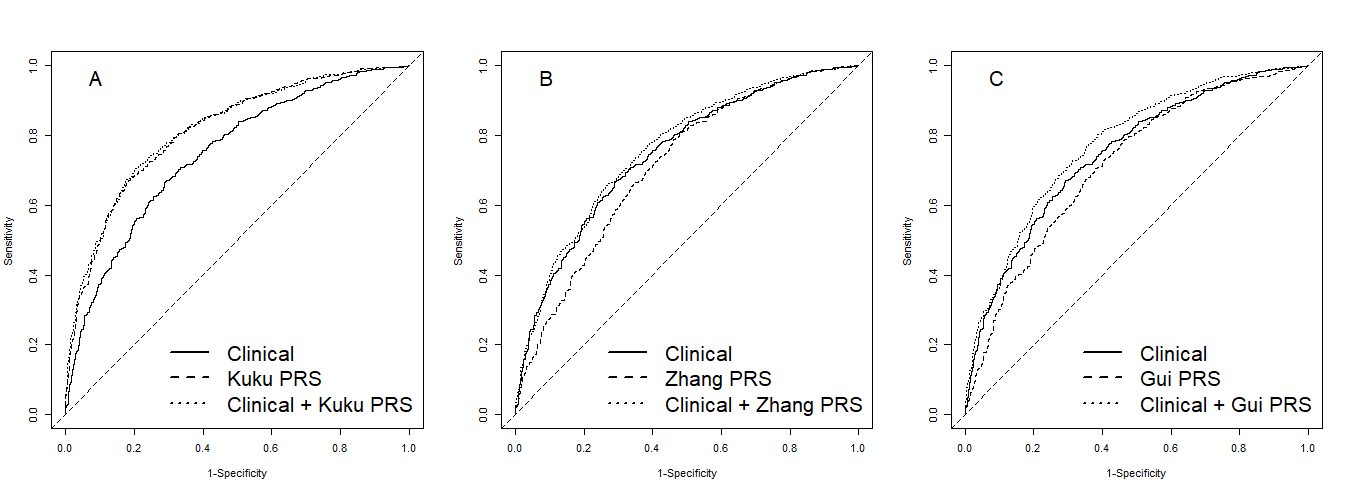
**

**S5 Fig.** Time-dependent AUC of 5-year mortality risk in community cohort by proteomics risk score stratified by ejection fraction.

**
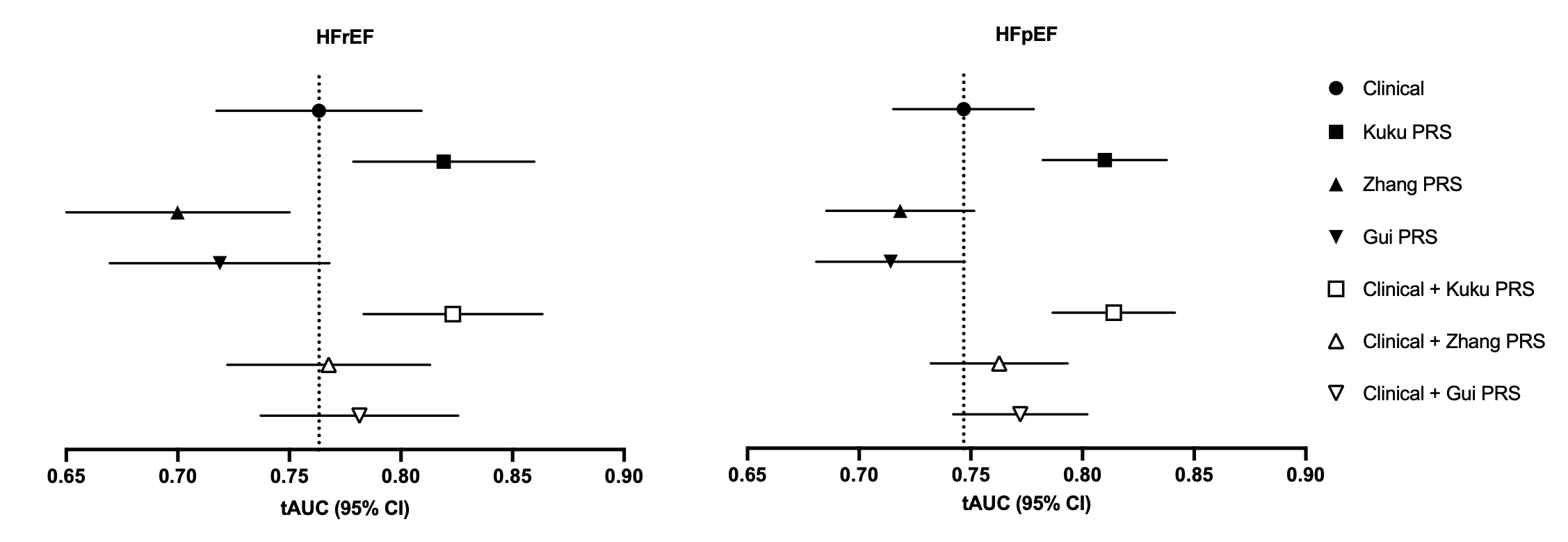
**

**S6 Fig.** Kaplan-Meier survival curves of three HF patient groups generated by K-means clustering using 7 common SOMAmers that were included in at least two of the three proteomics risk scores.


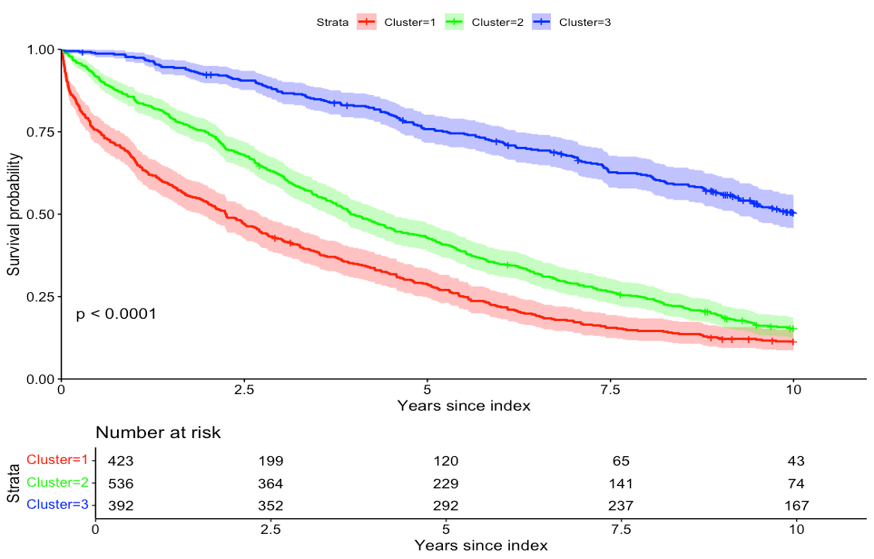

Supplement: S1 File — (DOCX) [file pone.0350697.s001.docx]
